# High-throughput screens to identify novel interactions between erythrocyte multi-pass receptors and *P. falciparum* merozoite surface ligands that are involved in invasion

**DOI:** 10.1186/1475-2875-9-S2-P57

**Published:** 2010-10-20

**Authors:** Madushi Wanaguru, Cecile Wright-Crosnier, Leyla Bustamante, Brian McDade, Julian Rayner, Gavin J Wright

**Affiliations:** 1 The Wellcome Trust Sanger Institute, Wellcome Trust Genome Campus, Hinxton, Cambridge, CB10 1SA, UK

## 

Invasion of erythrocytes by *P. falciparum* merozoites is a complex multistep process involving a series of molecular recognition events between parasite ligands and their receptors on the erythrocyte surface. Although many potential *P. falciparum* invasion ligands are known, the erythrocyte receptors they interact with have been identified only for a handful of cases. Studying extracellular binding events between membrane-embedded proteins *in vitro* is challenging and beyond the reach of standard approaches such as co-immunoprecipitation and yeast two-hybrid screens. These interactions are generally of very low affinity and often require specific motifs generated via post-translational processing; furthermore, amphipathic membrane-spanning proteins are difficult to manipulate biochemically. The Duffy Antigen Receptor for Chemokines (DARC), a multi-pass G-protein coupled erythrocyte receptor is known to be important for invasion by *P. vivax* but no multi-pass receptor has been established to play a role in *P. falciparum* invasion. With the aim of identifying such a receptor, we have developed a flow cytometry based, high-throughput strategy that can detect transient, extracellular protein: protein interactions. We are in the process of screening the soluble, recombinant ectodomains of 39 *P. falciparum* merozoite surface proteins against an expression library of 45 erythrocyte multi-pass surface proteins. We will present our screening strategy and preliminary results.

